# Headspace solid-phase microextraction coupled with gas chromatography-mass spectrometry (HS-SPME-GC-MS) and odor activity value (OAV) to reveal the flavor characteristics of ripened Pu-erh tea by co-fermentation

**DOI:** 10.3389/fnut.2023.1138783

**Published:** 2023-03-27

**Authors:** Yaru Zheng, Chunhua Zhang, Dabing Ren, Ruoxue Bai, Wenting Li, Jintao Wang, Zhiguo Shan, Wenjiang Dong, Lunzhao Yi

**Affiliations:** ^1^Faculty of Food Science and Engineering, Kunming University of Science and Technology, Kunming, Yunnan, China; ^2^College of Agriculture and Forestry, Pu’er University, Pu’er, Yunnan, China; ^3^Spice and Beverage Research Institute, Chinese Academy of Tropical Agricultural Sciences, Wanning, China

**Keywords:** ripened Pu-erh tea, flavor, mixed fermentation, HS-SPME-GC-MS, OAV

## Abstract

**Introduction:**

Pu-erh tea is a geographical indication product of China. The characteristic flavor compounds produced during the fermentation of ripened Pu-erh tea have an important impact on its quality.

**Methods:**

Headspace solid-phase microextraction coupled with gas chromatography-mass spectrometry (HS-SPME-GC-MS) and odor activity value (OAV) is used for flavor analysis.

**Results:**

A total of 135 volatile compounds were annotated, of which the highest content was alcohols (54.26%), followed by esters (16.73%), and methoxybenzenes (12.69%). Alcohols in ripened Pu-erh tea mainly contribute flower and fruit sweet flavors, while methoxybenzenes mainly contribute musty and stale flavors. The ripened Pu-erh tea fermented by *Saccharomyces*: *Rhizopus*: *Aspergillus niger* mixed in the ratio of 1:1:1 presented the remarkable flavor characteristics of flower and fruit sweet flavor, and having better coordination with musty and stale flavor.

**Discussion:**

This study demonstrated the content changes of ripened Pu-erh tea’s flavor compounds in the fermentation process, and revealed the optimal fermentation time. This will be helpful to further understand the formation mechanism of the characteristic flavor of ripened Pu-erh tea and guide the optimization of the fermentation process of ripened Pu-erh tea.

## 1. Introduction

As a beverage with health function, tea is widely welcomed. According to the degree of fermentation, tea is generally divided into green tea, yellow tea, white tea, oolong tea, black tea, and dark tea (1, 2). Ripened Pu-erh tea is one of the most popular dark teas (3). In recent years, many studies have demonstrated that Pu-erh tea produces unique flavor through microbial activities in the process of pile fermentation (4–9), which is considered as a key factor affecting the quality of dark tea (10).

The special sensory quality of ripened Pu-erh tea is one of the most important indicators of its market price. Some previous studies explored the main volatile compounds of ripened Pu-erh tea, and the results showed that the main flavors were 1,2,3-trimethoxybenzene, 1,2,4-trimethoxybenzene, hexadecanoic acid, dihydroactinidiolide, and so on, having stale, waxy or fruit flavor (4, 11, 12). And it was reported that during the pile fermentation, the compounds with flower flavor, such as phenylethyl alcohol, oxidized linalool, and linalool gradually decreased, and compounds with stale flavor, such as 1,2,3-trimethoxybenzene and 1,2,4-trimethoxybenzene gradually increased (13, 14). This makes the ripened Pu-erh tea exhibit a typical “aged fragrance.”

The dominant microorganisms are the key factors in the pile fermentation process of ripened Pu-erh tea. Previous studies have shown that the dominant species of ripened Pu-erh tea are mainly *Aspergillus*, *Penicillium*, and *Pseudolambica* (7, 15, 16). *Aspergillus* has been identified as the main flavor-producing microorganism (17). Studies have confirmed that specific microorganisms, such as *Aspergillus niger*, can improve the sensory quality of the tea by fermentation (18, 19). And there are studies on pile fermentation by inoculation of crown *Eurotium cristatum*, *Aspergillus niger*, and *Rhizopus* to affect volatile compounds and produce unique flower and fruit flavors (20, 21). So far, most of the existing studies focus on a few flavors and the content changes before and after fermentation, while systematic studies on various types of flavors in the fermentation process of ripened Pu-erh tea are rarely reported.

Headspace solid-phase microextraction (HS-SPME) coupled with gas chromatography-mass spectrometry (GC-MS) is a powerful technology to characterize the volatile compounds of tea (22, 23). HS-SPME-GC-MS is valuable for the characterization of tea flavor and allows a more comprehensive annotation of various volatile compounds in tea (24). However, the contribution of different volatile compounds to the flavor is very different, the annotation of volatile compounds is far from enough to reveal the flavor components in tea. Odor activity value (OAV) is the ratio of the concentration of flavor active compound to their flavor threshold value, which can help to identify the key flavor compounds in ripened Pu-erh tea (25). In general, compounds with OAV > 1 are considered as the main contributors to flavor (26–28).

In this study, Yunnan big-leaf sun-dried green tea (SGT) was fermented by 6 mixed strains, respectively, to obtain the ripened Pu-erh tea with flavor characteristics of flower and fruit sweet flavor. HS-SPME-GC-MS combined with OAV was employed to detect and reveal the flavor compounds of the ripened Pu-erh teas during the fermentation process. This study will help to reveal the changes of flavor compounds of ripened Pu-erh tea during fermentation, and provide valuable information for the optimization of ripened Pu-erh tea processing technology.

## 2. Materials and methods

### 2.1. Chemicals

N-alkanes, chromatographic pure grade (C_8_–C_40_) (o2si smart solutions Corporation)^1^. Decanoic acid ethyl ester, 98% purity (Sigma-Aldrich^2^).

### 2.2. Preparation of tea samples

Yunnan big-leaf sun-dried green tea (SGT) is used as the raw material for the pile fermentation of ripened Pu-erh tea. Each pile is stacked with 300 kg of SGT at 40% tide. Adding different proportions of beneficial strains to each pile of SGT, with 0.1% (w/w) of receiving bacteria. Group A, *Saccharomyces*: *Rhizopus* = 1:2; Group B, *Saccharomyces*: *Aspergillus niger* = 1:2; Group C, *Saccharomyces*: *Aspergillus oryzae* = 1:2; Group D, *Saccharomyces*: *Rhizopus*: *Aspergillus oryzae* = 1:1:1; Group E, *Saccharomyces*: *Rhizopus*: *Aspergillus niger* = 1:1:1; Group F, *Saccharomyces*: *Rhizopus*: *Aspergillus niger*: *Aspergillus oryzae* = 1:1:1:1. During the fermentation process, the pile was turned at the right time according to the changes in temperature, and humidity of the fermentation pile as well as the fermentation environment. The temperature, humidity, and pH of the tea pile were recorded at three time periods each day: 9:00 a.m., 15:00 p.m., and 21:30 p.m. Then the tea samples were taken on the 7th, 14th, 21st, 28th, and 35th days of fermentation, respectively, using the five-point sampling method, and the fermentation samples from the upper (10 cm thick), middle (30 cm thick), and lower (5 cm above the ground) parts were combined into one mixed sample. A total of 30 samples were collected in different mixed strains and different fermentation times in the pile (Supplementary Table 1). All samples collected are dried, ground into powder, placed in sealed bags (labeled with weight, time, and type), and stored in a −20°C refrigerator.

### 2.3. Extraction of volatile compounds in Pu-erh tea samples by HS-SPME

Headspace solid-phase microextraction was used to extract and enrich the volatile compounds in samples. The extraction fiber head type was 50/30 μm DVB/CAR/PDMS (SPME-GC Jeong-Jung Analytical Instruments Co). Accurately weigh 0.5 g of tea powder into a 20 mL headspace flask, 1.8 g of NaCl was added, and 10 μL of decanoic acid ethyl ester (0.2 mg/mL) was added as an internal standard. The headspace flask was sealed immediately after adding 5 mL of boiling water and the extraction fiber was inserted at 80°C for 40 min. After the extraction was completed, the solid-phase microextraction fiber needle was removed, and then inserted into the GC injection port for desorption (5 min, 260°C). To prevent contamination, the extracted fibers need to be aged at 270°C for 1 h at the GC-MS inlet before using. The above operation was repeated for each sample to minimize errors.

### 2.4. GC-MS analysis of volatile compounds

A combination of GC-MS (QP2010 Shimadzu, Japan) and HP-5MS quartz capillary column (30 m × 0.25 mm × 0.25 μm) was used. The inlet temperature was 260°C. The carrier gas was high purity helium (>99.999%) at a flow rate of 1 mL/min. The samples were taken in a split-flow injection with a split ratio of 5:1. The column temperature was set at 50°C and increased (ramped) at a rate of 10°C/min to 80°C; then increased at a rate of 3°C/min to 90°C for 3 min; then increased at a rate of 3°C/min to 120°C for 3 min; continued at a rate of 3°C/min to 170°C; and finally increased at a rate of 15°C/min to 230°C for 4 min.

Mass spectrometry (MS) conditions: The ion source was an EI source with an electron ionization energy of 70 eV. The interface temperature was 260°C and the ion source temperature was 230°C. And the mass range was 30–540 atomic mass unit (amu), solvent delay time of 3.0 min, full scan mode. The retention indices (RIs) was determined using a mixture of n-alkanes (C_8_–C_40_) running under the same conditions.

### 2.5. Qualitative and quantitative analysis of volatile compounds

#### 2.5.1. Qualitative analysis

The National Library of Standards and Technology (NIST) spectral library was used to search for compounds with >80% similarity, combined with C_8_–C_40_ n-alkanes to calculate RIs, and finally compared with the online database NIST Chemistry WebBook.^3^ The formula for calculating the retention indices is as follows,


$$R\,I\,s=100\,n+\frac{100\,\left(t_{i}-t_{n}\right)}{t_{n+1}-t_{n}}$$

Where *t*_*i*_ is the retention time of the compound to be measured, *t*_*n*_ and *t_*n*+1_* are the retention times of the mixture of n-alkane standards with n and n+1 carbon atoms, respectively, (*t_*n*_* < *t*_*i*_ < *t_*n*+1_*) (29).

#### 2.5.2. Quantitative analysis

Semi-quantitative analysis was performed using decanoic acid ethyl ester as an internal standard with the following formula,


$${\text{W}}_{\text{i}}=\frac{{\text{A}}_{\text{i}}}{{\text{A}}_{\text{s}}}\,{\text{W}}_{\text{s}}$$

Where W*_*i*_* is the content of the compound to be measured (μg/kg), A*_*i*_* is the peak area of the compound to be measured, A*_*s*_* is the peak area of the internal standard in the sample, and W*_*s*_* is the concentration of the internal standard (μg/kg) (30).

### 2.6. Odor activity values calculation

The thresholds of different volatile compounds in water (μg/kg) were obtained from the literatures (Supplementary Table 3), and then the OAV of each compound were calculated based on the quantitative results. The OAV calculated from the relative concentrations of the internal standard, which we define as relative odor activity values (rOAVs). The specific calculation formula is rOAVs = C*_*i*_*/OT*_*i*_*, where C*_*i*_* is the relative content of the compound by internal standard and OT*_*i*_* is its odor threshold in water. When rOAVs ≥ 1, it means that the compound has a large contribution to the flavor of the sample (31–33).

### 2.7. Statistical analysis

The experimental data were imported into the website for PCA analysis^4^ to distinguish between different mixed strains and the variability of volatile compounds during pile fermentation. Flavor characteristics of volatile compounds in Pu-erh tea were determined by literatures and websites,^5^ and to construct radar maps for compounds with rOAVs > 1 by Origin2022.

## 3. Results and discussion

### 3.1. Analysis of volatile components in ripened Pu-erh tea fermented by mixed strains

**FIGURE 1:**
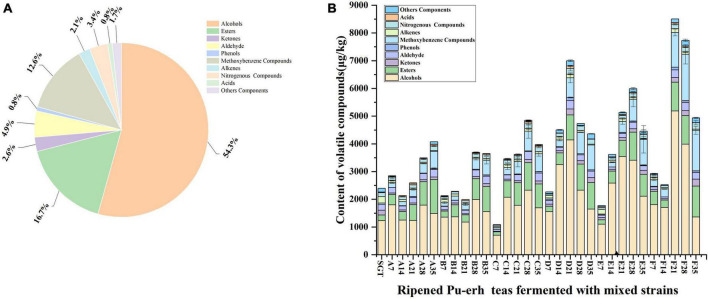
Contents of various volatile compounds. **(A)** Average percentage of various volatile compounds during the fermentation by different mixed strains; **(B)** flavor compounds of ripened Pu-erh tea during the fermentation of different mixed strains. A total of 7, 14, 21, 28, and 35 represent the fermentation time. SGT is the sun-dried green tea, A for *Saccharomyces*: *Rhizopus* = 1:2, B for *Saccharomyces*: *Aspergillus niger* = 1:2, C for *Saccharomyces*: *Aspergillus oryzae* = 1:2, D for *Saccharomyces*: *Rhizopus*: *Aspergillus oryzae* = 1:1:1, E for *Saccharomyces*: *Rhizopus*: *Aspergillus niger* = 1:1:1, F for *Saccharomyces*: *Rhizopus*: *Aspergillus niger*: *Aspergillus oryzae* = 1:1:1:1.

In this study, volatile compounds were detected by HS-SPME-GC-MS. A total of 135 compounds were annotated with the help of similarity research of online databases NIST Chemistry WebBook, and retention indices. As shown in Figure 1A, which were mainly divided into nine categories, including 34 alcohols (54.34%), 17 esters (16.68%), 14 ketones (2.62%), 11 aldehydes (4.88%), 6 phenols (0.76%), 14 methoxybenzenes (12.64%), 4 acids (0.84%), 9 alkenes (2.11%), 3 nitrogenous compounds (3.42%), and 23 others (1.69%). The number of volatile compounds detected in different samples is shown in the Supplementary Table 2, and the types and contents are shown in the Supplementary Table 3. The total contents of volatile compounds in Group D, E, and F reached 6,999.26 μg/kg, 5,132.07 μg/kg, and 7,264.87 μg/kg, respectively, when the fermentation proceeded to the 21st day, which was significantly higher than those in Group A, B, and C. This may be due to the increase in the abundance of fermentation strains. On the 35th day of pile fermentation, the content of total volatile compounds increased significantly compared to SGT, increasing 70.26, 52.71, 66.13, 82.17, 86.35, and 106.15% for the six groups, respectively. The results indicated that the type and total content of volatile compounds increased with the increasing of the diversity of mixed strains. Figure 1B shows that the total content of methoxybenzene, which has a stale and musty flavor, increased in ripened Pu-erh tea, which increased with the fermentation time and the abundance of fermenting mixed strains. Alcohol compounds were significantly higher in groups D, E, and F than in groups A, B, and C when fermented to the 21st day. Ester compounds increased significantly in their content with the increase of fermentation time. These differences in volatile compounds may be due to the differences in fermentation strains. During the pile-fermentation process, microorganisms secrete large amounts of peroxidases, cellulase, pectinase, lipase, and various hydrolases, which are involved in the oxidation, degradation, and molecular modification of catechins, gallic acid, and other aromatic precursors (3).

**FIGURE 2:**
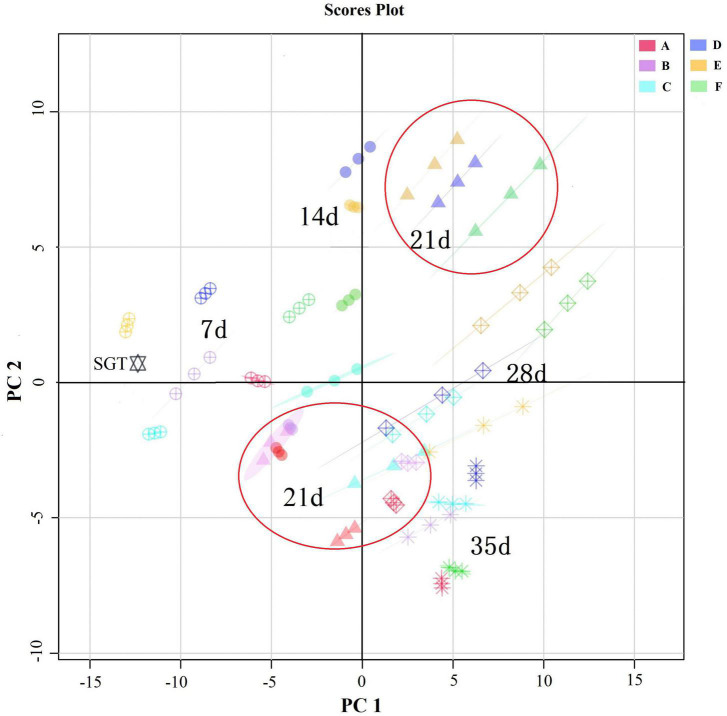
Comparison of the dynamics of ripened Pu-erh teas’ volatile compounds during mixed fermentation with different strains by PCA analysis. A total of 7 days, 14 days, 21 days, 28 days, and 35 days represents the fermentation time. SGT is the sun-dried green tea. A for *Saccharomyces*: *Rhizopus* = 1:2, B for *Saccharomyces*: *Aspergillus niger* = 1:2, C for *Saccharomyces*: *Aspergillus oryzae* = 1:2, D for *Saccharomyces*: *Rhizopus*: *Aspergillus oryzae* = 1:1:1, E for *Saccharomyces*: *Rhizopus*: *Aspergillus niger* = 1:1:1, F for *Saccharomyces*: *Rhizopus*: *Aspergillus niger*: *Aspergillus oryzae* = 1:1:1:1.

As shown in Figure 2, the results of principal component analysis (PCA) showed that the differences in volatile compounds increased with the increasing of fermentation time. The differences in volatile compounds at adjacent fermentation times were small. When fermentation proceeded to the 21st day, groups D, E, and F were mainly distributed in the first quadrant and groups A, B, and C were mainly distributed in the third quadrant, and their differences were significant. There were significant differences in the volatile compounds of the samples fermented with different mixed strains in the same fermentation time.

### 3.2. Flavor characteristics of ripened Pu-erh tea fermented with mixed strains

**FIGURE 3:**
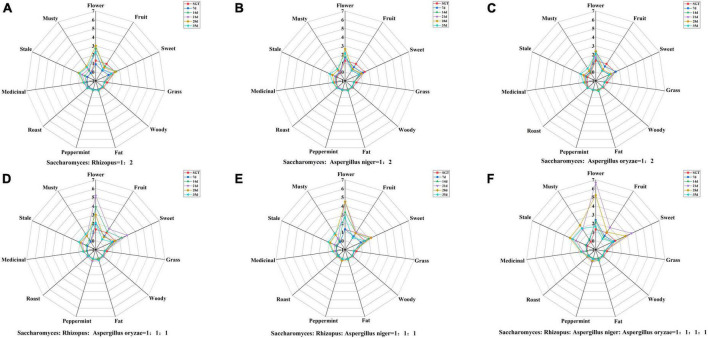
Radar plots of flavor characteristics of ripened Pu-erh teas during mixed fermentation with different strains (rOAVs > 1). **(A)** *Saccharomyces*: *Rhizopus* = 1:2; **(B)** *Saccharomyces*: *Aspergillus niger* = 1:2; **(C)** *Saccharomyces*: *Aspergillus oryzae* = 1:2; **(D)** *Saccharomyces*: *Rhizopus*: *Aspergillus oryzae* = 1:1:1; **(E)** *Saccharomyces*: *Rhizopus*: *Aspergillus niger* = 1:1:1; **(F)** *Saccharomyces*: *Rhizopus*: *Aspergillus niger*: *Aspergillus oryzae* = 1:1:1:1.

The contribution of volatile compound to tea flavor is not only related to the compound content but also to the odor threshold. Currently, OAV is commonly used to evaluate the contribution of volatile compounds to tea flavor, and it is generally believed that the larger the OAV, the greater contribution to the flavor, and compounds with OAV > 1 are usually considered as important flavor compounds in tea (34, 35). rOAVs values of volatile compounds of ripened Pu-erh tea fermented with mixed strains were shown in Table 1. There were 27 compounds with rOAVs > 1, including 8 alcohols, 1 ester, 4 ketones, 8 aldehydes, 3 methoxybenzenes, 2 alkenes, and 1 acid, such as linalool and its oxidation, geraniol, 6-methyl-5-hepten-2-one, 3,5-octadiene-2-ketone, 1,2,3-trimethoxybenzene, β-myrcene, limonene, and so on. In order to reveal the flavor characteristics of ripened Pu-erh teas, radar plots were constructed with the aroma characteristics of the 27 compounds (Figure 3). Radar plots of samples in the pile fermentation process from 7 to 35 days were shown in Supplementary Figure 1. At the end of fermentation on the 35th day, the ripened Pu-erh teas fermented with different mix strains was obtained. Figure 3 indicated that ripened Pu-erh teas of Group A, B, and C presented the flavor characteristics of flower and sweet, compared with SGT. Compared with Group A, B, and C, the flavor characteristics of Group E were more prominent. These ripened Pu-erh teas (Group A, B, C, and E) present typical flower, fruit, and sweet flavor (Figure 3), which was defined as flower and fruit sweet flavor. The flower intensity of group D was similar to that of groups A, B, and C but the musty, stale and medicinal flavors were stronger than those of the three groups (Figure 3D, 35 days). F was significantly different from the first five groups, and this group had significantly higher musty and stale flavors than the other groups after 35 days of fermentation, and the flower flavor was significantly weaker than that other groups. This group of ripened Pu-erh tea presented typical stale and musty flavor (Figure 3F, 35 days). Previous studies (12) have indicated that *Aspergillus* is the main glucosidase-producing genus, and the fermentation treatment by microorganisms can effectively increase terpene alcohols from glycosides and linalool oxides through oxidation, and the increasing in the content of these compounds contributes to the formation of ripened Pu-erh tea with flower and fruit sweet flavor.

**TABLE 1:** Flavor compounds’ rOAVs of ripened Pu-erh teas fermented by mixed strains.

No.	Flavor compound	OT (μg/kg)^a^	Odor description^b^	rOAVs										
				**SGT**	**A7**	**A14**	**A21**	**A28**	**A35**	**B7**	**B14**	**B21**	**B28**	**B35**
1	1-Octen-3-ol	1	Grass	158.89	25.77	15.88	10.55	17.99	13.39	23.81	14.74	14.62	14.05	13.81
2	Linalool oxide I	60	Flower	1.12	2.61	3.23	3.27	5.26	5.10	1.88	3.51	3.01	5.87	4.51
3	Linalool	6	Flower, Fruit, and Sweet	52.56	54.31	17.16	16.74	22.15	12.17	42.65	30.23	21.75	28.08	13.29
4	Phenylethyl alcohol	4	Flower	30.57	120.31	77.94	66.89	105.67	93.38	85.48	72.38	61.00	119.94	91.22
5	Linalool oxide II	320	Flower	0.08	0.28	0.41	0.59	0.74	0.70	0.12	0.43	0.44	0.76	0.91
6	Terpinen-4-ol	0.2	Woody	71.75	55.58	27.36	n.d.	32.61	n.d.	57.93	36.90	30.38	39.59	n.d.
7	α-Terpineol	350	Sweet	0.48	0.72	0.33	0.30	0.40	0.33	0.57	0.45	0.36	0.52	0.36
8	Geraniol	7.5	Flower, Fruit, and Sweet	9.52	9.35	2.90	3.46	4.97	3.09	7.69	4.28	3.58	3.75	2.28
9	Methyl salicylate	40	Peppermint	0.56	0.43	0.58	0.54	0.94	0.83	0.42	0.46	0.39	1.33	1.03
10	6-methyl-5-Hepten-2-one	68	Fruit	1.04	0.15	0.09	0.10	0.15	0.17	0.14	0.09	0.09	0.22	0.24
11	3,5-Octadien-2-one	0.5	Fruit and Fat	34.77	n.d.	10.49	23.31	20.28	33.49	n.d.	n.d.	n.d.	24.04	24.02
12	α-Ionone	0.4	Flower	10.75	6.18	3.73	n.d.	n.d.	n.d.	n.d.	n.d.	n.d.	n.d.	n.d.
13	β-Ionone	0.2	Flower, Fruit, and Woody	164.58	79.27	55.69	78.12	70.73	79.32	82.41	74.62	61.24	71.34	70.67
14	2-Heptenal	20	–	0.85	0.48	0.37	n.d.	0.65	n.d.	n.d.	n.d.	0.38	n.d.	n.d.
15	2,4-Heptadienal	2.56	Fat	8.97	5.10	5.46	14.10	12.30	21.29	4.65	4.29	4.92	10.47	15.38
16	Benzeneacetaldehyde	4	Flower and Sweet	5.72	5.42	3.81	5.88	5.65	5.66	4.11	3.40	3.95	6.37	4.94
17	1H-Pyrrole-2-carboxaldehyde,1-ethyl	2	Roast	39.38	57.18	39.85	40.17	48.85	56.84	38.25	27.37	28.19	61.18	64.03
18	2-Octenal	3	Fat	2.52	1.10	n.d.	1.31	n.d.	2.93	n.d.	n.d.	n.d.	1.26	2.00
19	Nonanal	1	Fruit, Flower, and Fat	n.d.	n.d.	n.d.	18.02	17.92	18.65	n.d.	n.d.	13.68	15.77	16.08
20	2,6-Nonadienal	0.1	Fruit and Flower	n.d.	n.d.	n.d.	n.d.	n.d.	99.24	n.d.	n.d.	n.d.	n.d.	78.89
21	Decanal	0.1	Sweet and Fruit	26.37	19.93	21.77	44.82	26.65	48.83	19.86	n.d.	16.31	19.57	24.00
22	Benzene, 1,2-dimethoxy	3.17	Sweet and Musty	5.30	10.16	10.30	18.20	22.89	32.12	6.66	11.95	11.49	24.91	22.42
23	1,2,3-Trimethoxybenzene	0.75	Stale and Musty	29.04	91.31	57.42	147.39	153.96	318.89	64.45	69.32	92.85	194.74	322.33
24	1,2,4-Trimethoxybenzene	3.06	Stale and Medicinal	2.75	20.64	19.87	29.68	47.96	67.85	9.68	13.48	27.39	47.86	68.94
25	β-Myrcene	13	Woody, Fruit, and Peppermint	2.99	1.24	1.62	0.83	0.65	0.63	2.78	0.81	0.66	0.72	0.70
26	D-Limonene	10	Sweet and Fruit	10.38	3.87	3.18	2.81	2.45	n.d.	4.63	3.99	2.81	2.94	3.15
27	Nonanoic acid	1.5	Fat and Sweet	17.34	13.89	11.66	21.50	10.74	18.36	8.24	5.95	13.87	10.75	14.36
**No.**	**Compound**	**OT** **(μg/kg)^a^**	**Odor description^b^**		**rOAVs**									
					**C7**	**C14**	**C21**	**C28**	**C35**	**D7**	**D14**	**D21**	**D28**	**D35**
1	1-Octen-3-ol	1	Grass		8.23	22.06	28.83	19.01	15.12	45.26	21.94	47.27	18.65	24.36
2	Linalool oxide I	60	Flower		0.97	4.82	4.79	6.98	5.35	2.70	8.18	14.36	8.61	8.44
3	Linalool	6	Flower, Fruit, and Sweet		19.82	51.72	29.19	39.19	20.64	46.35	84.06	99.86	35.56	14.71
4	Phenylethyl alcohol	4	Flower		48.96	118.30	96.40	112.84	80.66	111.36	152.51	128.11	93.81	42.52
5	Linalool oxide II	320	Flower		0.08	0.57	0.76	0.96	0.92	0.16	0.65	1.36	0.94	0.54
6	Terpinen-4-ol	0.2	Woody		23.75	62.87	35.16	50.32	n.d.	53.06	97.66	105.45	50.93	37.21
7	α-Terpineol	350	Sweet		0.28	0.67	0.52	0.74	0.44	0.46	1.00	1.07	0.62	0.45
8	Geraniol	7.5	Flower, Fruit, and Sweet		4.94	8.34	3.32	4.55	2.61	6.72	14.38	15.68	3.86	2.61
9	Methyl salicylate	40	Peppermint		0.22	0.52	1.07	0.96	1.12	1.19	1.65	4.31	1.77	1.08
10	6-methyl-5-Hepten-2-one	68	Fruit		0.05	0.15	0.13	0.16	0.16	0.26	0.24	0.55	0.21	0.29
11	3,5-Octadien-2-one	0.5	Fruit, Fat		n.d.	n.d.	13.06	21.25	21.18	14.43	23.87	54.63	43.09	60.88
12	α-Ionone	0.4	Flower		2.70	7.35	n.d.	8.97	n.d.	8.80	11.84	15.34	8.91	8.59
13	β-Ionone	0.2	Flower, Fruit, and Woody		37.58	94.34	n.d.	108.00	86.95	96.36	95.50	197.75	109.95	104.79
14	2-Heptenal	20	–		n.d.	n.d.	0.69	0.70	0.63	1.01	0.63	1.56	0.80	0.79
15	2,4-Heptadienal	2.56	Fat		1.97	5.99	10.34	12.28	13.93	8.54	7.11	27.96	19.38	27.83
16	Benzeneacetaldehyde	4	Flower and Sweet		1.96	4.91	4.82	6.09	5.46	4.57	3.96	6.09	4.68	3.19
17	1H-Pyrrole-2-carboxaldehyde,1-ethyl	2	Roast		21.18	44.48	53.18	65.68	68.18	15.76	26.94	57.51	33.39	31.28
18	2-Octenal	3	Fat		0.49	n.d.	1.88	1.46	1.66	2.96	n.d.	4.77	3.24	2.61
19	Nonanal	1	Fruit, Flower, and Fat		n.d.	n.d.	14.84	19.82	16.23	n.d.	n.d.	n.d.	16.69	17.95
20	2,6-Nonadienal	0.1	Fruit and Flower		n.d.	n.d.	n.d.	n.d.	n.d.	n.d.	n.d.	n.d.	0.00	n.d.
21	Decanal	0.1	Sweet and Fruit		n.d.	n.d.	21.55	31.37	24.85	17.50	n.d.	50.97	0.00	n.d.
22	Benzene, 1,2-dimethoxy	3.17	Sweet and Musty		3.69	24.17	24.23	37.16	32.49	7.82	31.28	83.38	62.19	79.99
23	1,2,3-Trimethoxybenzene	0.75	Stale and Musty		25.21	158.20	285.84	479.80	420.69	23.64	71.53	238.80	326.31	412.44
24	1,2,4-Trimethoxybenzene	3.06	Stale and Medicinal		5.91	19.00	47.81	70.16	63.57	2.31	10.59	56.30	70.05	75.56
25	β-Myrcene	13	Woody, Fruit, and Peppermint		0.61	1.33	0.89	1.01	0.81	0.92	1.59	1.90	0.77	0.52
26	D-Limonene	10	Sweet and Fruit		2.17	5.73	4.08	5.15	4.64	3.18	4.35	5.64	3.24	n.d.
27	Nonanoic acid	1.5	Fat and Sweet		8.23	12.19	10.44	12.66	10.53	6.00	n.d.	22.80	0.00	n.d.
**No.**	**Compound**	**OT** **(μg/kg)^a^**	**Odor description^b^**		**rOAVs**									
					**C7**	**C14**	**C21**	**C28**	**C35**	**D7**	**D14**	**D21**	**D28**	**D35**
1	1-Octen-3-ol	1	Grass		49.45	17.76	23.80	16.99	15.69	29.85	12.28	37.47	19.36	28.61
2	Linalool oxide I	60	Flower		2.36	7.45	12.37	13.27	8.89	4.51	5.18	17.82	16.67	7.72
3	Linalool	6	Flower, Fruit, and Sweet		40.41	76.09	80.60	68.49	33.63	51.65	39.10	113.65	74.73	16.04
4	Phenylethyl alcohol	4	Flower		41.67	106.35	137.66	113.38	58.31	104.49	85.24	177.96	121.42	26.49
5	Linalool oxide II	320	Flower		0.10	0.60	1.05	1.35	0.87	0.43	0.55	2.14	1.64	0.26
6	Terpinen-4-ol	0.2	Woody		52.98	67.05	80.86	n.d.	53.10	54.30	41.42	103.00	65.90	32.06
7	α-Terpineol	350	Sweet		0.33	0.81	1.01	0.92	0.59	0.49	0.47	1.33	1.04	0.31
8	Geraniol	7.5	Flower, Fruit, and Sweet		5.95	13.11	12.73	9.95	3.90	4.96	4.97	18.16	9.02	1.35
9	Methyl salicylate	40	Peppermint		1.20	1.60	3.35	3.54	1.18	0.78	0.57	5.35	3.94	1.57
10	6-methyl-5-Hepten-2-one	68	Fruit		0.33	0.16	0.35	0.26	0.19	0.18	0.09	0.42	0.28	0.45
11	3,5-Octadien-2-one	0.5	Fruit and Fat		17.06	16.18	22.10	28.98	42.51	13.11	12.66	39.18	52.85	84.76
12	α-Ionone	0.4	Flower		9.27	7.83	9.31	12.16	8.34	10.11	4.68	19.61	12.24	7.97
13	β-Ionone	0.2	Flower, Fruit, and Woody		101.92	75.95	84.77	91.89	79.74	84.74	49.00	167.99	159.72	121.76
14	2-Heptenal	20	–		0.53	0.42	0.75	0.65	0.44	0.51	0.28	0.96	0.62	0.75
15	2,4-Heptadienal	2.56	Fat		7.85	5.58	10.94	17.65	17.41	6.22	4.30	18.85	25.00	33.38
16	Benzeneacetaldehyde	4	Flower and Sweet		3.82	2.67	3.11	3.82	3.30	n.d.	1.64	5.06	4.57	3.25
17	1H-Pyrrole-2-carboxaldehyde,1-ethyl	2	Roast		3.89	17.46	32.64	46.03	24.80	15.72	14.01	67.92	55.22	25.46
18	2-Octenal	3	Fat		1.90	n.d.	2.32	2.82	2.26	1.60	0.73	3.45	3.33	3.42
19	Nonanal	1	Fruit, Flower, and Fat		n.d.	n.d.	n.d.	17.36	15.02	n.d.	n.d.	0.00	23.38	n.d.
20	2,6-Nonadienal	0.1	Fruit and Flower		n.d.	n.d.	n.d.	123.73	n.d.	n.d.	n.d.	0.00	142.07	119.20
21	Decanal	0.1	Sweet and Fruit		n.d.	n.d.	n.d.	n.d.	38.07	n.d.	n.d.	70.08	49.99	29.92
22	1,2-dimethoxybenzene	3.17	Sweet and Musty		4.66	24.66	51.07	81.21	83.33	27.88	32.23	109.81	123.86	106.39
23	1,2,3-Trimethoxybenzene	0.75	Stale and Musty		14.92	49.81	136.58	342.72	440.54	89.52	112.41	711.75	1025.16	814.02
24	1,2,4-Trimethoxybenzene	3.06	Stale and Medicinal		1.28	4.83	23.70	60.90	71.30	8.63	13.01	71.87	115.13	110.59
25	β-Myrcene	13	Woody, Fruit, and Peppermint		1.97	1.12	1.28	1.04	0.72	1.18	0.62	2.45	1.03	0.44
26	D-Limonene	10	Sweet and Fruit		4.30	3.28	4.41	3.94	n.d.	3.55	2.42	6.27	4.89	n.d.
27	Nonanoic acid	1.5	Fat and Sweet		12.80	6.54	12.50	11.32	n.d.	n.d.	n.d.	24.72	16.63	n.d.

OT, odor threshold; n.d., not detected; 7, 14, 21, 28, and 35, fermentation time; SGT, sun-dried green tea.

^a^Threshold value of different volatile compounds in water (μg/kg).

^b^Odor description for the volatile compounds.

A for *Saccharomyces*: *Rhizopus* = 1:2; B for *Saccharomyces*: *Aspergillus niger* = 1:2; C for *Saccharomyces*: *Aspergillus oryzae* = 1:2; D for *Saccharomyces*: *Rhizopus*: *Aspergillus oryzae* = 1:1:1; E for *Saccharomyces*: *Rhizopus*: *Aspergillus niger* = 1:1:1; F for *Saccharomyces*: *Rhizopus*: *Aspergillus niger*: *Aspergillus oryzae* = 1:1:1:1.

### 3.3. Changes of flower and fruit sweet flavor chemicals during fermentation process

**FIGURE 4:**
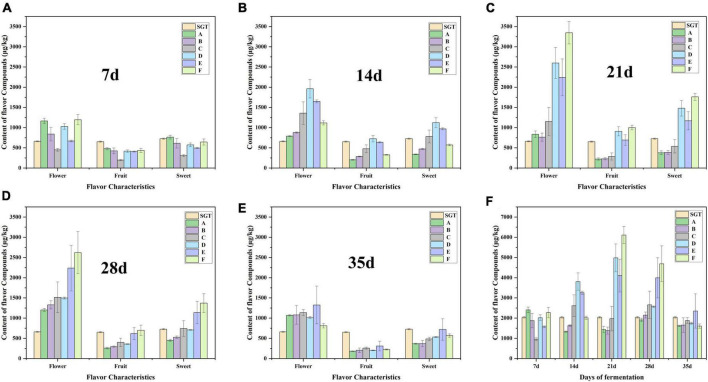
Content changes of the 17 volatile compounds with flower, fruit or sweet flavor during the fermentation process. Panels **(A–E)** are the content changes from 7 to 35 days, respectively. Panel **(F)** is the change in total content of the 17 compounds.

Here we focus on the changes of chemicals with flower and fruit sweet flavor in Group D, E, and F. This research can help us find the suitable fermentation time of Pu-erh tea with pleasant flavor. There are 17 compounds having flower and fruit sweet flavor, including linalool, geraniol, 6-methyl-5-hepten-2-one, 3,5-octadien-2-one, limonene, linalool oxide, phenylethyl alcohol, α-Ionone, β-Ionone, benzeneacetaldehyde, nonanal, 2,6-nonadienal, decanal, β-myrcene, nonanoic acid, α-terpineol. Linalool presents flower, sweet, and fruit flavors (35, 36). Compounds that have a fruit flavor mainly include 6-methyl-5-hepten-2-one, 3,5-octadien-2-one, and limonene, the sweet compounds are mainly benzeneacetaldehyde, and the compounds that are mostly flower flavor are linalool oxide, geraniol, phenylethyl alcohol, and α-Ionone (26, 35–38). β-Ionone is a key aromatic compound in tea and contributes flower and fruit flavor (35, 37, 38). Nonanal and 2,6-nonadienal have fruit and flower flavor. Nonanoic acid and α-terpineol have sweet flavor. Decanal and β-myrcene have sweet and fruity flavor. The flavor profiles of these compounds were checked from http://www.thegoodscentscompany.com/search3.php. The total contents of these flavor compounds reached its maximum on the 21st day (Figure 4F), especially the flower flavor compounds (Figure 4C). The flower and fruit sweet flavor of Group D, E, and F increased significantly at the 21st day compared with the sun-dried green tea, by 144.27, 101.20, and 199.59%, respectively. When the fermentation continued to 35 days, the flower and fruit sweet flavor of Group E was stronger than that of SGT, increasing by 15.36%, decreasing 20.70, 18.80, 7.87, 14.33, and 21.32% in Group A, B, C, D, and F, respectively (Figure 4).

**FIGURE 5:**
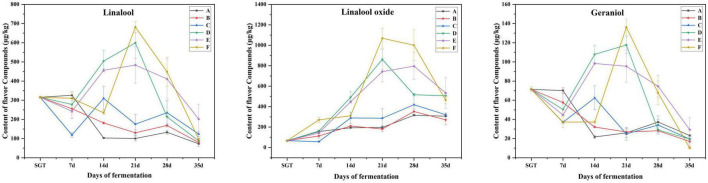
Content changes of linalool, linalool oxide, and geraniol with flower flavor during the fermentation process. A for *Saccharomyces*: *Rhizopus* = 1:2, B for *Saccharomyces*: *Aspergillus niger* = 1:2, C for *Saccharomyces*: *Aspergillus oryzae* = 1:2, D for *Saccharomyces*: *Rhizopus*: *Aspergillus oryzae* = 1:1:1, E for *Saccharomyces*: *Rhizopus*: *Aspergillus niger* = 1:1:1, F for *Saccharomyces*: *Rhizopus*: *Aspergillus niger*: *Aspergillus oryzae* = 1:1:1:1.

The three main flower flavor compounds mainly include linalool, linalool oxide, and geraniol, all increased significantly when the fermentation proceeded to the 21st day, then gradually deceased (Figure 5). Microorganisms can release β-primeverosides and β-glucopyranosides through enzymatic hydrolysis, and then biosynthesize linalool, and its oxides. It can increase the content of linalool and its oxides in this way (12). In summary, in this study, the flower and fruit sweet flavor of the ripened Pu-erh tea fermented for 21 days is the most remarkable, which is fermented by *Saccharomyces*: *Rhizopus*: *Aspergillus niger* mixed in the ratio of 1:1:1 (Group E).

### 3.4. Characterization of methoxybenzenes in ripened Pu-erh tea fermented by mixed strains

Methoxybenzenes are typical flavor compounds in ripened Pu-erh tea, which present stale flavor (39, 40). However, high contents of methoxybenzenes usually have stale and musty flavor (9, 25, 41, 42, 43).

**FIGURE 6:**
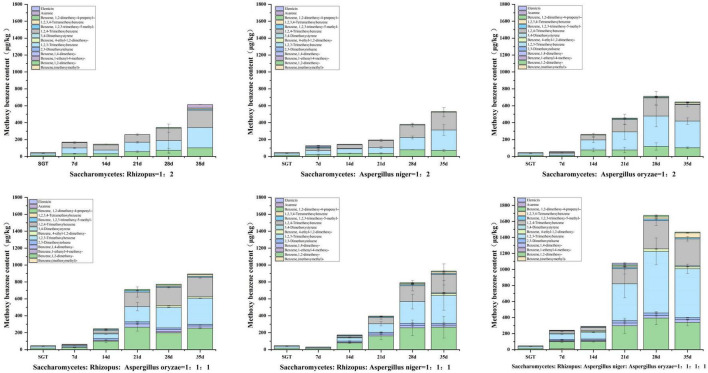
Content changes of 14 methoxybenzenes during the fermentation process.

The total of 14 methoxybenzenes were annotated in this study, including 1,2-dimethoxybenzene, 1,2,3-trimethoxybenzene, 1,2, 4-trimethoxybenzene, 1,4-dimethoxybenzene, 2,3-dimethoxytol- uene, 3,4,5-trimethoxytoluene, 1,2,3,4-tetramethoxybenzene, 1,2-dimethoxy-4-propenyl-benzene, elemicin, asarone, et al. It was found that the types and contents of methoxyphenols produced in Group A, B, and C were lower than those in Group D, E, and F (Figure 6). The total content of methoxybenzenes reached to 613.60 μg/kg, 531.66 μg/kg, and 644.44 μg/kg in Group A, B, and C at the 35th day of fermentation, respectively. The total content of methoxybenzenes reached to 893.42 μg/kg and 927.88 μg/kg for Group D and E, respectively, by 35 days of fermentation. Group E had better coordination between flower and fruit sweet flavor and musty and stale flavor, compared with Group D. The total content of methoxybenzenes in group F reached 1,466.20 μg/kg by 35 days of fermentation, which was significantly higher than the other groups. In this study, it was found that the contents of methoxybenzenes increased with the prolongation of fermentation time, and the species and contents increased with the increase of strain richness, as shown in Figure 6. It is noteworthy that many reports described the odor of methoxybenzenes such as 1,2,3-trimethoxybenzene and 1,2,4-trimethoxybenzene as musty and stale, which may make methoxybenzenes contribute musty and stale flavor to the ripened Pu-erh tea (39). Furthermore, previous investigation showed that microorganisms can increase the content of gallic acid (GA) by degrading epigallocatechin gallate (EGCG) and hydrolyzing tannins during the fermentation of ripened Pu-erh tea in a hot and humid environment. Microorganisms such as *Aspergillus niger* can replace the hydrogen atoms of hydroxyl radicals in GA with methyl groups, resulting in methoxybenzenes, and compounds with similar structures (8, 12). Similar to our results, the pile fermentation process may contribute to the accumulation of methoxybenzenes.

## 4. Conclusion

In this study, ripened Pu-erh tea with flower and fruit sweet flavor was obtained by *Saccharomyces*, *Rhizopus*, and *Aspergillus niger* co-fermentation, using Yunnan big-leaf sun-dried green tea as fermentation raw materials. With the help of HS-SPME-GC-MS, a total of 135 volatile compounds were annotated, and due to the different strains and fermentation time, the ripened Pu-erh teas’ volatile compounds varied greatly. OAV analysis illustrated that there were 17 volatile compounds presenting flower and fruit sweet flavor. The total content of these compounds increased until the 21th day and then decreased, which indicated that the 21th day was an important time point for the fermentation of ripened Pu-erh tea with flower and fruit sweet flavor. There were 14 volatile compounds showing musty and stale flavor. The content of these compounds increased with the extension of fermentation time, and the types and contents increased with the abundance of the strains. The flavor of ripened Pu-erh tea is the result of the synergistic effect of different flavor compounds. In this study, the flavor characteristics and content changes of volatile components in ripened Pu-erh tea during fermentation were demonstrated in detail. This will help us to further understand the formation mechanism of the characteristic flavor of ripened Pu-erh tea, so as to guide the optimization of the fermentation process of ripened Pu-erh tea.

## Data availability statement

The original contributions presented in this study are included in the article/Supplementary material, further inquiries can be directed to the corresponding authors.

## Author contributions

LY was responsible for methodology, writing—review, commentary editing, supervision, resources, and funding acquisition. WD was responsible for resources, supervision, methodology, and writing—review. YZ was responsible for writing—original draft, experimentation, formal analysis, and methodology. CZ and ZS were responsible for resources. DR was responsible for the formal analysis. RB, WL, and JW were responsible for investigation. All authors contributed to the article and approved the submitted version.

## Footnotes

1
http://www.vicbio.com/Index/show/catid/22/id/403.html

2
https://www.sigmaaldrich.cn/CN/zh/campaigns/promotion/proclin-preservatives?utm_campaign=Brand%20Zone%20-%20China&utm_medium=Sigma-Aldrich_brandzone&utm_source=baidu&utm_content=headline&utm_term=proclin-preservatives

3
https://webbook.nist.gov/chemistry/name-ser/

4
https://www.metaboanalyst.ca/

5
http://www.thegoodscentscompany.com/search3.php
