# Supplementary material for: Headspace solid-phase microextraction coupled with gas chromatography-mass spectrometry (HS-SPME-GC-MS) and odor activity value (OAV) to reveal the flavor characteristics of ripened Pu-erh tea by co-fermentation
Source: Front Nutr. 2023 Mar 27;10:1138783. doi: 10.3389/fnut.2023.1138783 (PMC10083425; doi:10.3389/fnut.2023.1138783)
Supplement: Supplementary file 1 [file Presentation_1.pptx]

## Slide 1
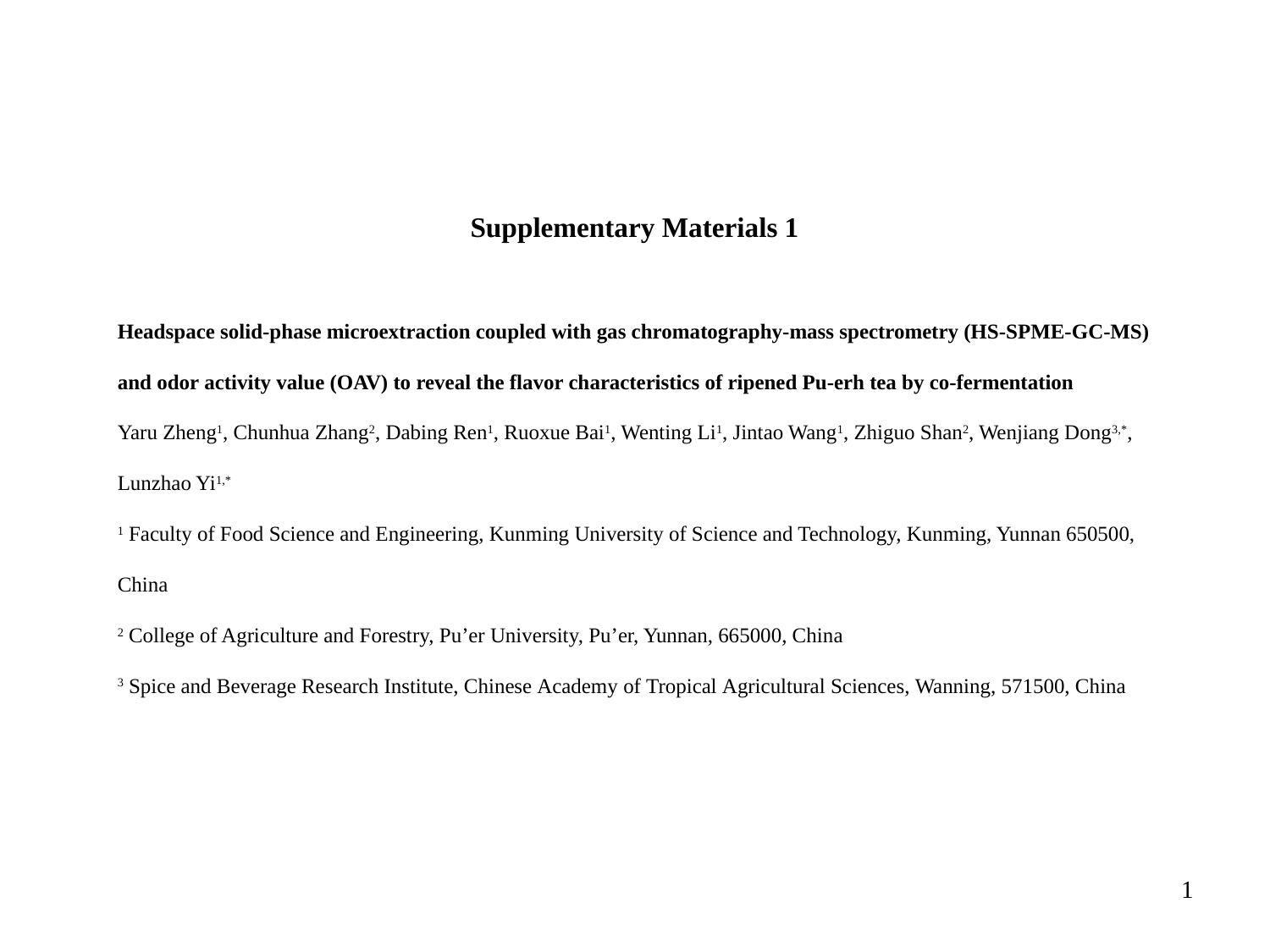

# Supplementary Materials 1
Headspace solid-phase microextraction coupled with gas chromatography-mass spectrometry (HS-SPME-GC-MS) and odor activity value (OAV) to reveal the flavor characteristics of ripened Pu-erh tea by co-fermentation
Yaru Zheng1, Chunhua Zhang2, Dabing Ren1, Ruoxue Bai1, Wenting Li1, Jintao Wang1, Zhiguo Shan2, Wenjiang Dong3,*, Lunzhao Yi1,*
1 Faculty of Food Science and Engineering, Kunming University of Science and Technology, Kunming, Yunnan 650500, China
2 College of Agriculture and Forestry, Pu’er University, Pu’er, Yunnan, 665000, China
3 Spice and Beverage Research Institute, Chinese Academy of Tropical Agricultural Sciences, Wanning, 571500, China
1

## Slide 2
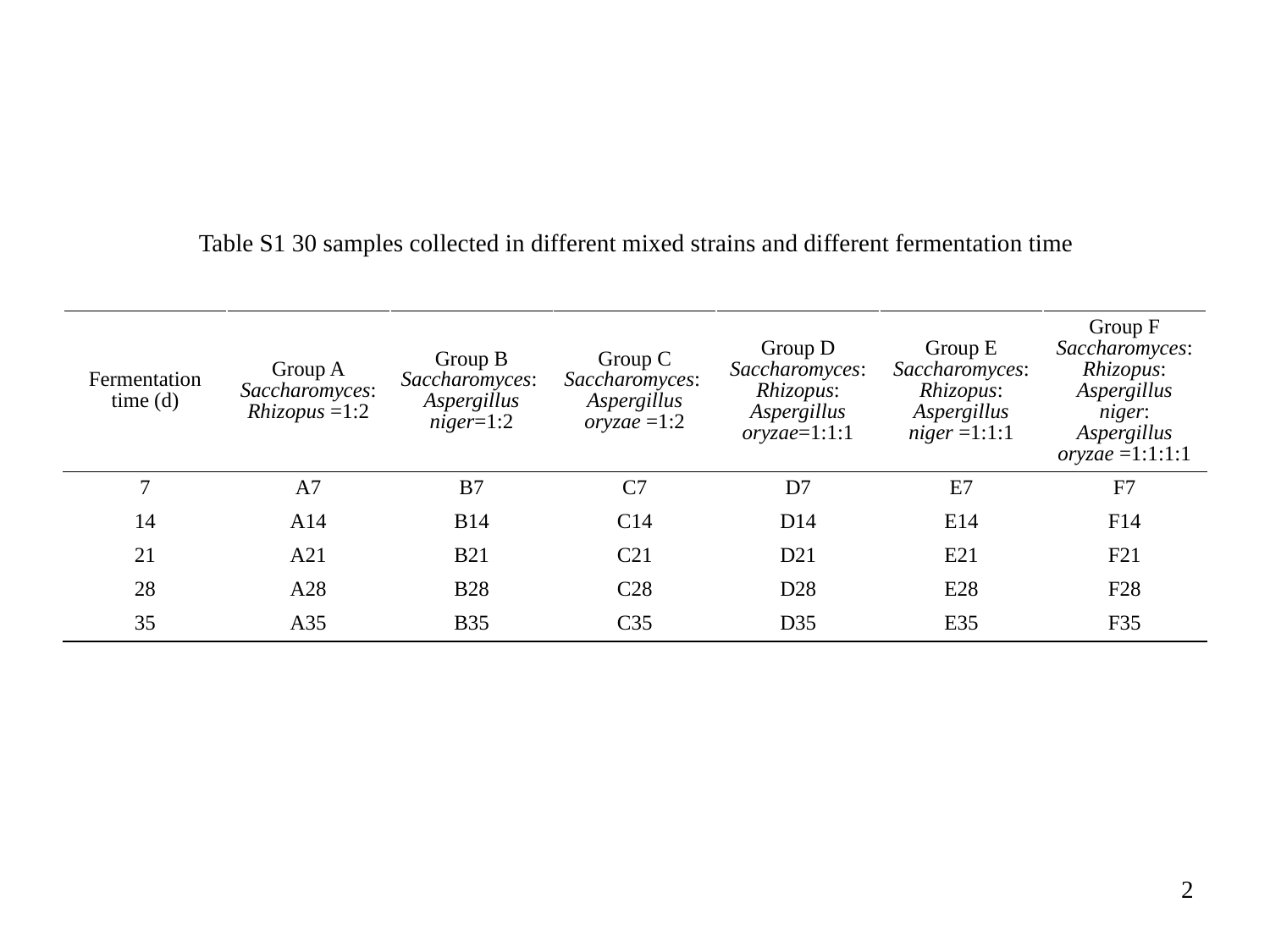

Table S1 30 samples collected in different mixed strains and different fermentation time
| Fermentation time (d) | Group A Saccharomyces: Rhizopus =1:2 | Group B Saccharomyces: Aspergillus niger=1:2 | Group C Saccharomyces: Aspergillus oryzae =1:2 | Group D Saccharomyces: Rhizopus: Aspergillus oryzae=1:1:1 | Group E Saccharomyces: Rhizopus: Aspergillus niger =1:1:1 | Group F Saccharomyces: Rhizopus: Aspergillus niger: Aspergillus oryzae =1:1:1:1 |
| --- | --- | --- | --- | --- | --- | --- |
| 7 | A7 | B7 | C7 | D7 | E7 | F7 |
| 14 | A14 | B14 | C14 | D14 | E14 | F14 |
| 21 | A21 | B21 | C21 | D21 | E21 | F21 |
| 28 | A28 | B28 | C28 | D28 | E28 | F28 |
| 35 | A35 | B35 | C35 | D35 | E35 | F35 |
2

## Slide 3
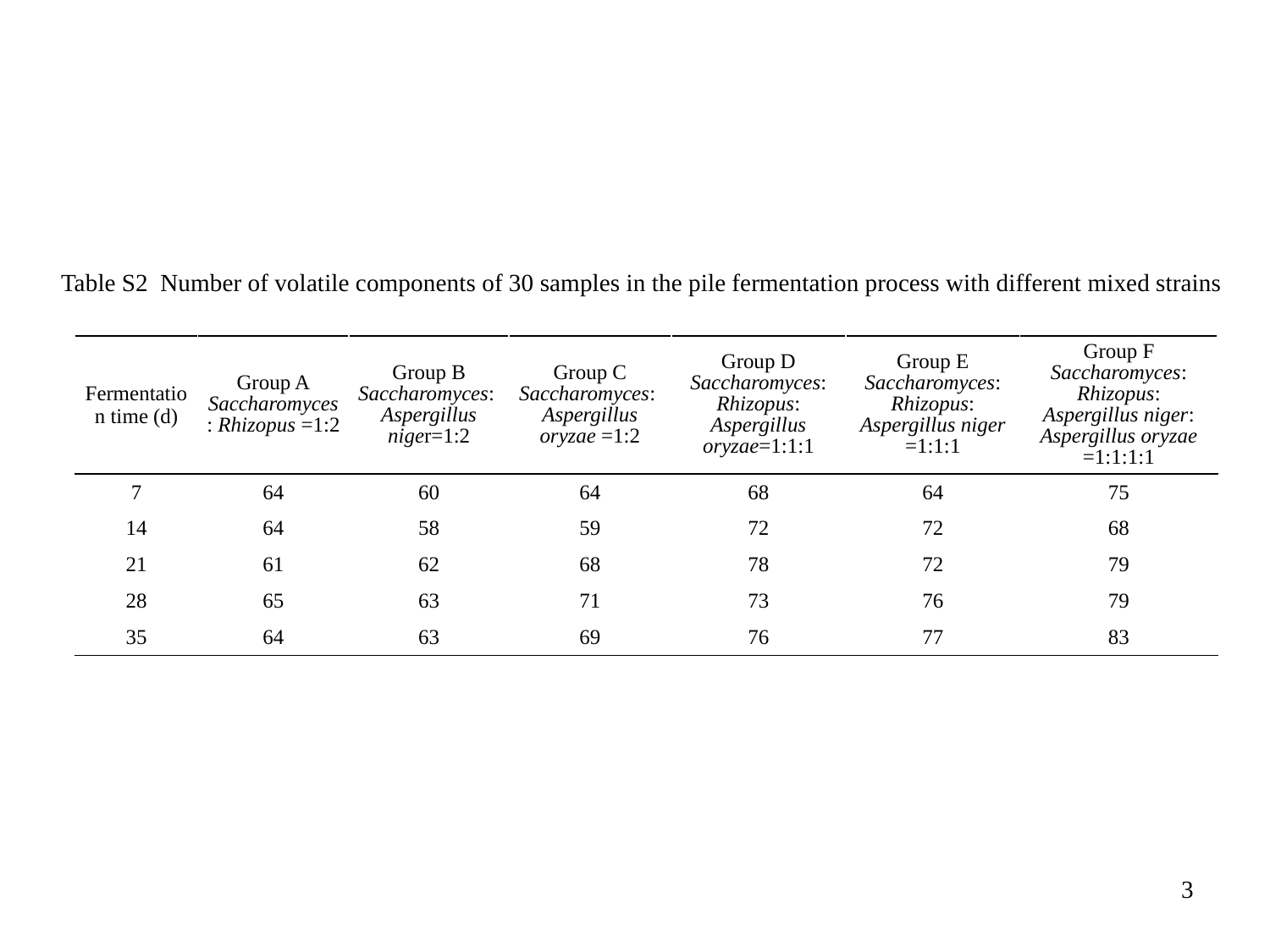

Table S2 Number of volatile components of 30 samples in the pile fermentation process with different mixed strains
| Fermentation time (d) | Group A Saccharomyces: Rhizopus =1:2 | Group B Saccharomyces: Aspergillus niger=1:2 | Group C Saccharomyces: Aspergillus oryzae =1:2 | Group D Saccharomyces: Rhizopus: Aspergillus oryzae=1:1:1 | Group E Saccharomyces: Rhizopus: Aspergillus niger =1:1:1 | Group F Saccharomyces: Rhizopus: Aspergillus niger: Aspergillus oryzae =1:1:1:1 |
| --- | --- | --- | --- | --- | --- | --- |
| 7 | 64 | 60 | 64 | 68 | 64 | 75 |
| 14 | 64 | 58 | 59 | 72 | 72 | 68 |
| 21 | 61 | 62 | 68 | 78 | 72 | 79 |
| 28 | 65 | 63 | 71 | 73 | 76 | 79 |
| 35 | 64 | 63 | 69 | 76 | 77 | 83 |
3

## Slide 4
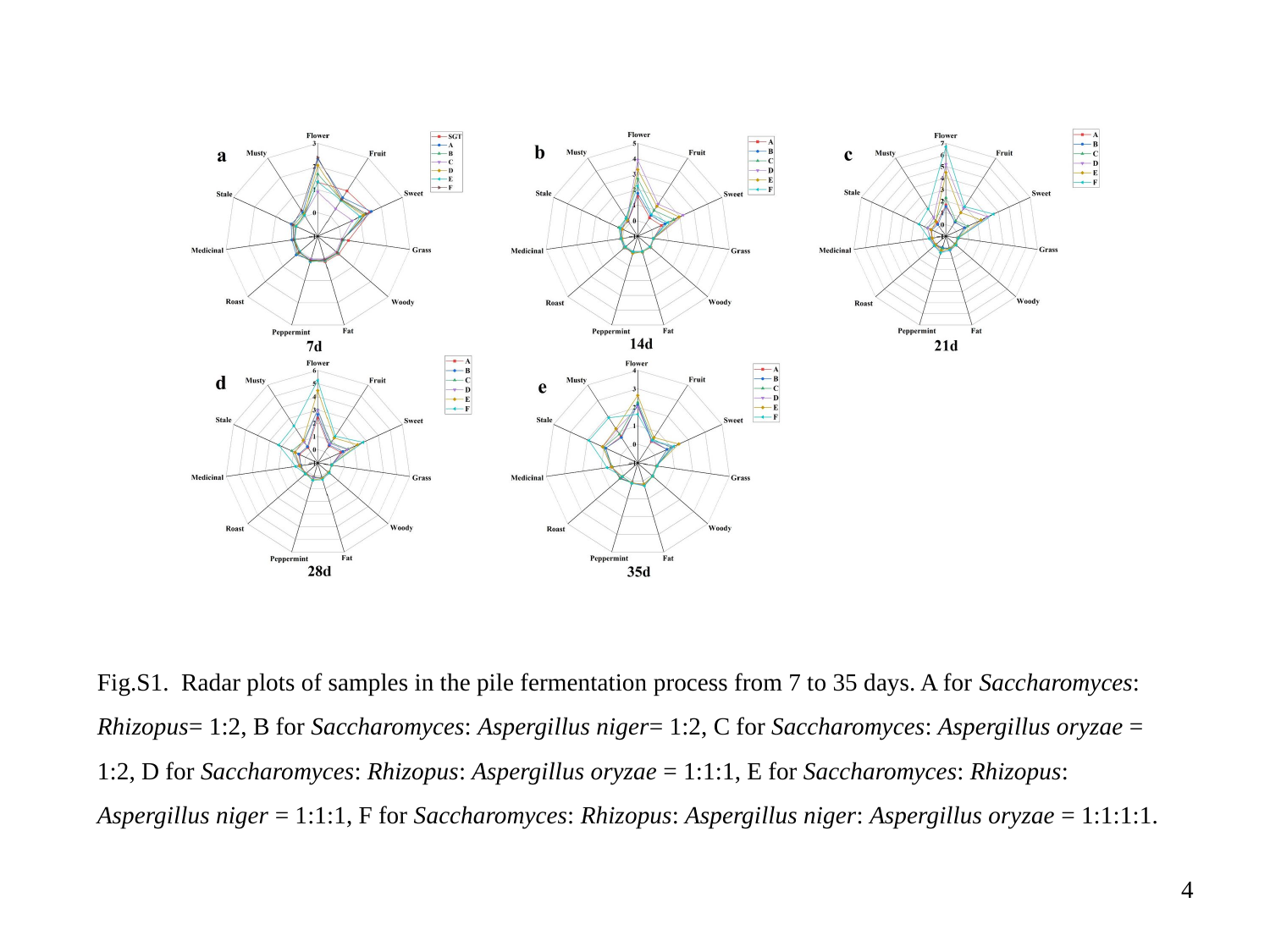

Fig.S1. Radar plots of samples in the pile fermentation process from 7 to 35 days. A for Saccharomyces: Rhizopus= 1:2, B for Saccharomyces: Aspergillus niger= 1:2, C for Saccharomyces: Aspergillus oryzae = 1:2, D for Saccharomyces: Rhizopus: Aspergillus oryzae = 1:1:1, E for Saccharomyces: Rhizopus: Aspergillus niger = 1:1:1, F for Saccharomyces: Rhizopus: Aspergillus niger: Aspergillus oryzae = 1:1:1:1.
4
